# Treatment outcomes and antiretroviral uptake in multidrug-resistant tuberculosis and HIV co-infected patients in Sub Saharan Africa: a systematic review and meta-analysis

**DOI:** 10.1186/s12879-019-4317-4

**Published:** 2019-08-16

**Authors:** Elvis Dzelamonyuy Chem, Marie Claire Van Hout, Vivian Hope

**Affiliations:** Public Health Institute, Liverpool John Moores University, Liverpool, UK

**Keywords:** Multidrug-resistant tuberculosis, MDR-TB and HIV co-infection, Treatment success, Unsuccessful treatment, Sub Saharan Africa

## Abstract

**Background:**

Multidrug-resistant tuberculosis (MDR-TB) in HIV endemic settings is a major threat to public health. MDR-TB is a substantial and underreported problem in Sub-Saharan Africa (SSA), with recognised cases projected to increase with advancement in diagnostic technology. There is paucity of review evidence on treatment outcomes and antiretroviral (ART) uptake among MDR-TB patients with HIV in SSA. To address this gap a review of treatment outcomes in HIV patients co-infected with MDR-TB in the SSA region was undertaken.

**Methods:**

Three databases (Medline, Web of Science, CINHAL), Union on Lung Heath conference proceedings and grey literature were searched for publications between January 2004 and May 2018. Records were assessed for eligibility and data extracted. Random effect meta-analysis was conducted using STATA and Cochrane’s review manager.

**Results:**

A total of 271 publications were identified of which nine fulfilled the inclusion criteria. Data was collected from 3368 MDR-TB and HIV co-infected patients from four SSA countries; South Africa (6), Lesotho (1), Botswana (1) and Ethiopia (1). The most common outcome was cure (34.9% cured in the pooled analysis), this was followed by death (18.1% in pooled analysis). ART uptake was high, at 83% in the pooled analysis. Cure ranged from 28.6 to 54.7% among patients on ART and from 22.2 to 57.7%  among those not on ART medication. MDR-TB and HIV co-infected patients were less likely to be successfully treated than HIV negative MDR-TB patients (Risk Ratio = 0.87, 95% CI 0.97, 0.96).

**Conclusion:**

Treatment outcomes for MDR-TB and HIV co-infected patients do not vary widely from those reported globally. However, treatment success was lower among HIV positive MDR-TB patients compared to HIV negative MDR-TB patients. Prompt antiretroviral initiation and interventions to improve treatment adherence are necessary.

**Electronic supplementary material:**

The online version of this article (10.1186/s12879-019-4317-4) contains supplementary material, which is available to authorized users.

## Background

Globally Tuberculosis (TB) remains a major cause of death, with drug resistant forms resurging over the past two decades [1]. Multidrug-resistant strains of TB (those resistant to at least rifampicin and isoniazid) (MDR-TB) have been associated with increased mortality and are a particular issue among those living with the human immunodeficiency virus (HIV) [2]. The 2017 Global TB report estimated 10 million new TB cases, 1.6 million deaths, with 0.3 million of these among those with HIV. The burden is more concentrated in Sub Saharan Africa (SSA) due to the high level of TB and HIV co-infection in this region, with over 51% of TB cases co-infected with HIV [3]. Globally, about 558,000 MDR-TB cases were diagnosed in 2017, an increase from the 490,000 cases in 2016 [3, 4]. Among the 490,000 MDR-TB cases in 2016, only 22% were initiated on treatment. Un-initiated cases, the majority of which are reported from Africa and Asia [4], constitute a significant reservoir of infection in the community. Considering the synergy between HIV and TB infections in SSA, many un-initiated MDR-TB cases will also be HIV coinfected [3].

Treatment provision for MDR-TB and HIV co-infection has been scaled-up over the past decade but remains below requirements in SSA [5]. This treatment gap reflects a range of interconnected issues including the complexity of MDR-TB, which includes long treatment duration (24 months) with large numbers of pills taken daily, invasive daily intramuscular injections, side effects due to drug toxicity [6] and complicities of the clinical management of the co-infection. This results in high mortalities and poor treatment outcomes, a phenomenon that has been described as the “perfect storm” [7, 8]. As a result of these complexities, there are a range of potential treatment outcomes and these are usually classified into: cure, treatment completed, death, treatment default, treatment failure, transfer out and treatment success [9, 10]. To improve outcomes during treatment of MDR-TB and HIV co-infection, the World Health Organization (WHO) recommends concurrent treatment with second line anti-tuberculosis drugs (SLD) and antiretroviral therapy (ART), irrespective of CD4 cell counts [11]. Following its roll out in SSA in 2004, ART has reduced mortality in MDR-TB and HIV co-infected patients, yet HIV in MDR-TB cases remains a significant predictor of death, with complications in management of MDR-TB and HIV co-infection leading to high mortality. Late ART initiation and health system factors such as timely diagnosis of drug resistance and lack of supportive financial systems have partly been blamed for this observation [12].

Research on treatment outcomes in SSA has assessed: the effect of ART initiation time on treatment outcomes [13, 14], treatment success and survival in MDR-TB and HIV co-infected cohorts [15–17] and predictors of outcomes [14]. Previous reviews have addressed the risk factors of HIV and MDR-TB in SSA [7, 18]. Yet, to date no review on treatment outcomes in MDR-TB by HIV status in SSA has been conducted. A global review on TB treatment outcomes in adults and children [6] reported no difference in treatment success between HIV positive and negative MDR-TB patients. We speculate that the finding in relation to HIV status may have been diluted by low HIV prevalence in other regions. The global review also included studies conducted prior to rollout of ART in SSA. Therefore, we undertook a review to investigate treatment outcomes for MDR-TB and HIV co-infected patients in SSA in the era of HIV ART to help inform policy, practice and research on the treatment of MDR-TB and HIV co-infection in SSA.

## Methods

This Prospero registered study (ID: CRD42018095600) followed the Preferred Reporting Items for Systematic Reviews and Meta-Analyses (PRISMA) guidelines.

### Data sources

We searched Medline (EbscoHost), Web of Science Core collections, CINAHL (EbscoHost) and Cochrane library for articles published between January 2004 (2004 was when roll out of ART across SSA started) and May 2018. The search strategy was modified from those used by Samuels et al. [19] and Isaakidis et al. [6]. The following Medical Subject Headings and free text terms were used; Tuberculosis, Multidrug-Resistant Tuberculosis, MDR-TB, HIV, Human Immunodeficiency Virus, People living with HIV, PLWH, HIV/TB co-infection, drug resistant tuberculosis/HIV coinfection, Multidrug resistant tuberculosis HIV coinfection, Sub Saharan Africa, Africa South of Sahara, Antiretroviral therapy, Antiretroviral medication, Antiretroviral regimen, Antiretroviral administration, Highly active antiretroviral, HAART, ART, ARV, Treatment, Treatment outcome, Survival, Success, Failure, default, cure, died, loss to follow up, treatment completed (see Additional file 7).

To identify studies only in grey literature, abstracts submitted to the annual conference of the International Journal of Tuberculosis and Lung disease (IJTLD) from 2005 to 2017 were manually searched. Authors whose abstracts matched inclusion criteria were contacted via e-mail for additional data. Annual reports and publications by Medecins Sans Frontieres, WHO, United Nations Joint Commission on HIV/AIDS (UNAIDS) and US Presidents Emergency Plan for AIDS Relief (PEPFAR) were also searched.

### Inclusion criteria

Included studies fulfilled the following characteristics: 1) Included culture or drug susceptibility testing confirmed MDR-TB patients 2) clearly reported the use of either individualized, standardized or mixed MDR-TB treatment regimen, 3) reported the use of ART during the treatment of MDR-TB and HIV co-infection 4) reported at least one of the six outcomes recommended by Learson et al and WHO [9, 10], 5) documented a minimal age of 15 years for study participants, 6) conducted between January 2004 when ART roll out started to May 2018 (with the exception of studies that compared MDR-TB, and MDR-TB and HIV co-infected patients that began with collection of MDR-TB data earlier than 2004 and subsequently incorporated MDR-TB and HIV co-infection data from 2004), and 7) conducted in SSA, and published in English.

### Study selection

After exhaustive database, bibliographic and manual searching, retrieved studies were screened with duplicates removed using the EndNote X8 reference management software. Publication titles and abstracts were initially screened, and non-relevant ones excluded. The full text of retained studies were read and those that did not match inclusion criteria were excluded with justification. Study quality and risk of bias in reporting findings was assessed. Two authors (EDC) and (VH) independently conducted article and abstract screening, while the third author (MCVH) validated these. Disagreements on articles to include/exclude were resolved through consensus.

### Data extraction

We extracted data using a data extraction form, designed in Microsoft Excel based on those used in other reviews and piloted on five studies. Data was extracted by one author and checked by another. In cases of disagreement, a consensus was reached among all authors. Key data extracted for each study include: year of publication, study design, study country, study description, sample size, participant age, number with confirmed MDR-TB and HIV co-infection, number with MDR-TB, number on ART & time of initiation on ART, type of MDR-TB regimen, MDR-TB treatment duration, treatment setting (centralised hospital-based treatment or decentralised community, clinic and hospital- based treatment), treatment outcomes, and predictors of MDR-TB treatment failure and mortality (see Additional file 8).

### Quality assessment

Cohort and case control studies were assessed for quality using the Newcastle-Ottawa quality assessment scale [20] and the randomized control trial using the National Institute of Health (NIH), quality assessment tool for controlled intervention studies [21]. Indicators of good study quality captured by both tools were; use of standardized method to confirm MDR-TB and HIV status; large sample size with a cut off value of at least 40 participants; multicentre study; integration of home, clinic and hospital based care; use of appropriate statistical tests to classify and report outcomes; taking confounders (demographics, socioeconomic status, previous treatment history) into account during data analysis; clear method of participant selection; and representativeness of study participants to the population of MDR-TB and HIV co-infected population. In addition, use of a valid source for retrieving outcomes and participant information; adequate treatment duration; reporting less than 1/3 missing data at final analysis compared to original population recruited, and proof of ethical review of the study were also considered.

Treatment outcomes were recorded following the proposal by Learson et al [9] and WHO [10] definitions (see Additional file 1).

### Data analysis and synthesis

STATA 13.1 (Statacorp) and Review manager version 5.3 were used for meta-analysis. Heterogeneity among studies was evaluated at 95% confidence level using binary effect analysis. To facilitate analysis and enable computation of dichotomous effect measures, outcomes were grouped into two categories; treatment success (cure and competed treatment) and unsuccessful treatment (death, defaulted, lost to follow up, failure). Studies were included in the analysis based on outcomes reported. A sub-group analysis for treatment success and unsuccessful treatment in those with MDR-TB according to HIV status was conducted using the Mantel-Haensel random effects method. Heterogeneity of binary covariates was estimated using I^2^ and *P* values, at 95% confidence level.

## Results

**Fig. 1:**
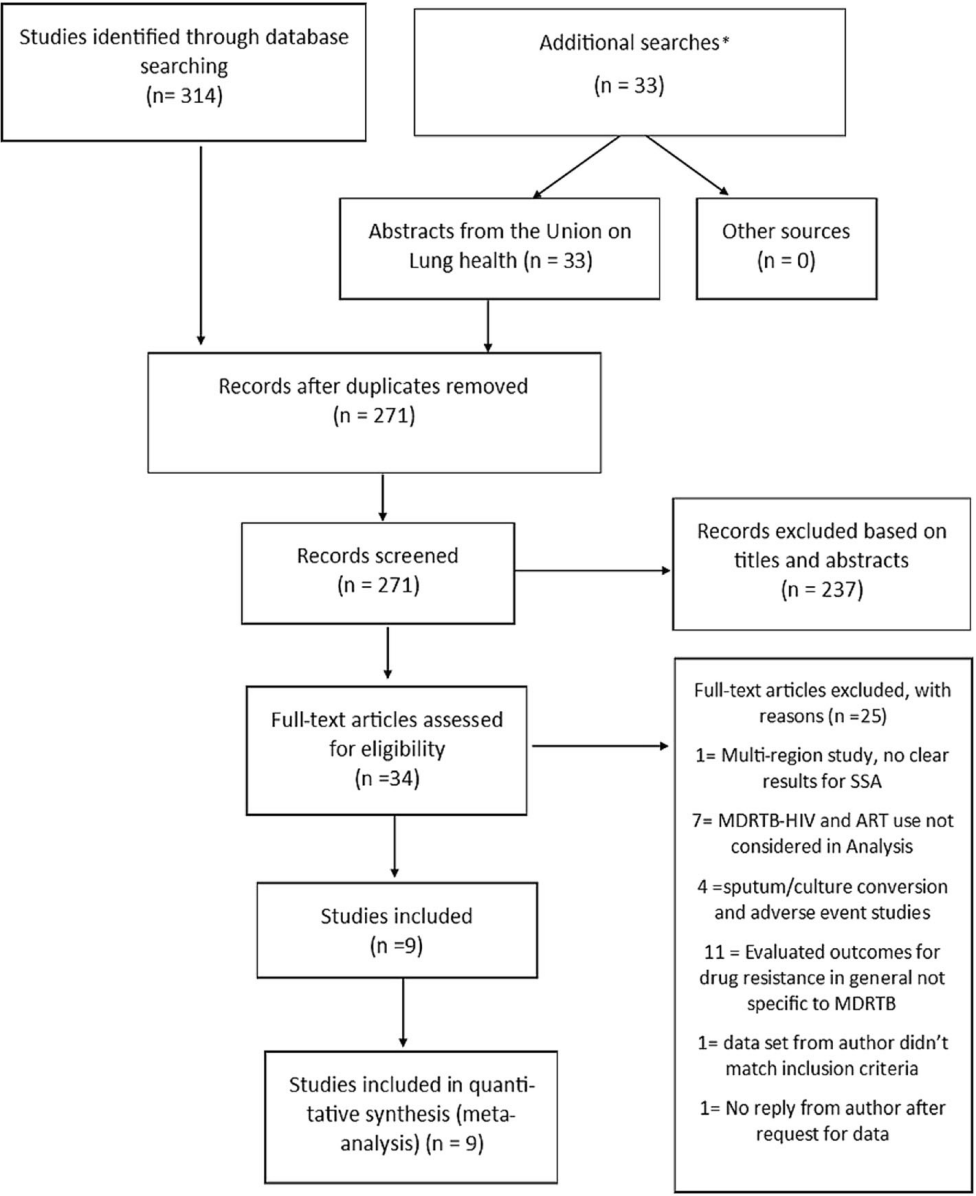
PRISMA flow chart illustrating selection of included studies from SSA on MDR-TB treatment in those with MDR-TB and HIV coinfection. *Reports and publications from World Health Organisation, Medecin Sans Frontieres, UNIAIDS, PEPFAR

Database searches identified 314 articles and 33 records were obtained from additional sources (all from the abstract books of the annual World Conference on Lung Health). Requests for additional data were sent to two authors and one responded. Following deduplication, there were 271 unique records. Titles and abstracts were screened excluding 237 of these. Full text of the remaining 34 were assessed, 25 were excluded (Fig. 1). Nine studies were thus included in this review.

### Study characteristics

The nine studies were published between 2012 and 2018. Their sample sizes ranged from 23 to 1137 (mean = 580) and they had a total of 4666 participants. Of these, 3368 were MDR-TB and HIV co-infected. Study duration varied from one year [13] to over eight years [16], with majority (*n* = 4) of the studies conducted over periods of three to four years [14, 22–24]. Six studies were conducted in South Africa [14, 16, 22–25], one in Lesotho [13], one in Botswana [17] and one in Ethiopia [15]. Five studies were retrospective cohort studies [13–16, 24], with two prospective cohort studies [17, 22], one case control study [23] and one randomized controlled trial (RCT) [25]. Six studies reported treatment for pulmonary and extra-pulmonary tuberculosis [13–15, 22, 24, 25]. However, three studies did not specify the type of MDR-TB treated [16, 17, 23]. Six studies were conducted in centralised MDR-TB treatment settings [14, 16, 22–25], while three were in a decentralised treatment setting [13, 15, 17]. Not all studies reported the type of anti-tuberculosis drug [16, 25] and ART used [14, 16, 17, 24] during treatment of MDR-TB and HIV co-infection. Treatment duration and follow-up for MDR-TB and HIV co-infected patients within the studies ranged between 18 and 24 months (see Additional file 2).

### Treatment outcomes

Cure was the most common outcome reported, ranging from 26.1 to 68% across the studies, with a pooled proportion cured 34.9% (see Additional file 3). One study reported the combined outcome for cure and completed treatment [16]. Deaths were the second most common outcome, pooled proportion 18.1%, study range 11.2 to 34.3%. Default was reported in six studies and ranged from 1 to 22.3%. The least commonly reported outcome was still being on treatment at the end of follow-up (see Additional file 4).

### ART uptake and treatment success

**Fig. 2:**
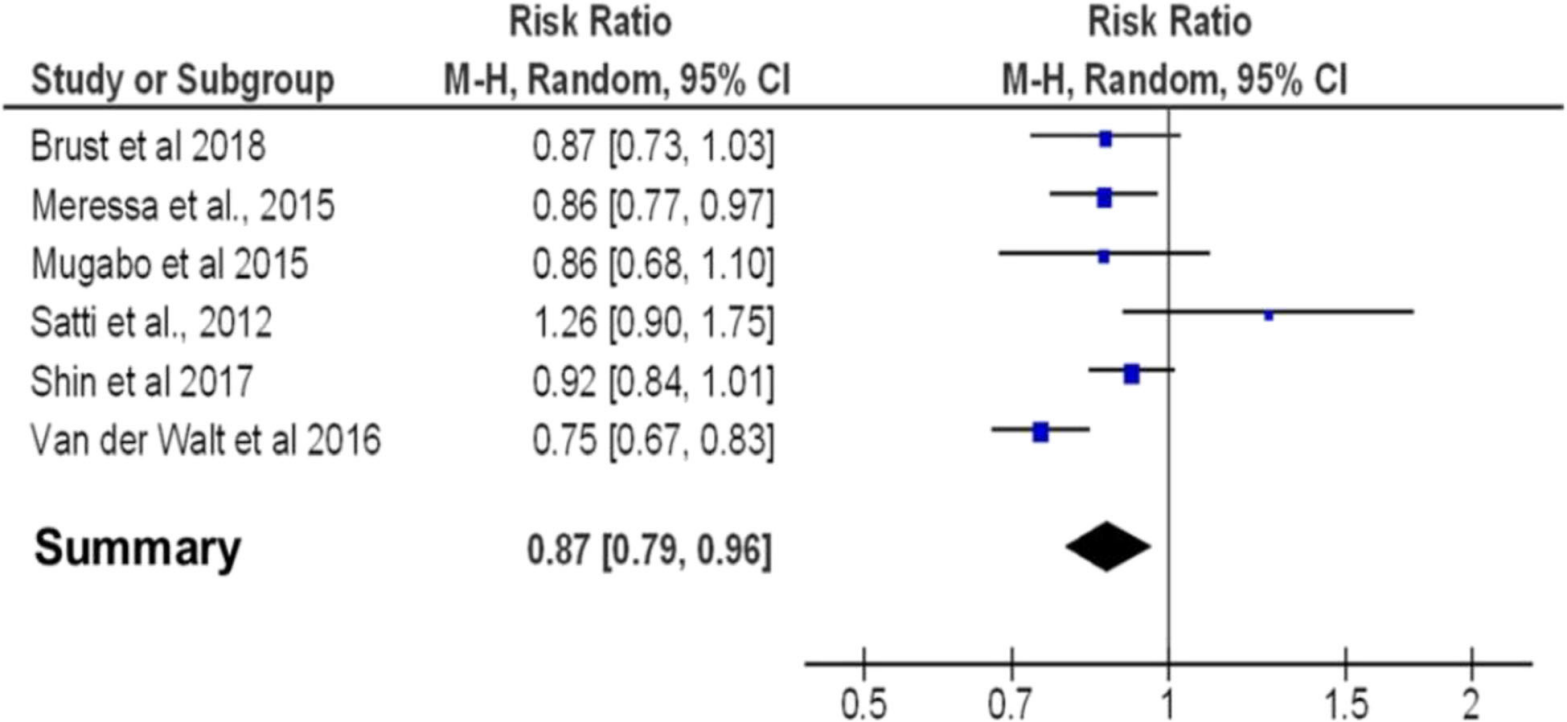
Forest plot of studies that quantitatively assessed the association between treatment success and HIV status among MDR-TB patients in SSA. CI = Confidence Interval MH = Mantel Hansel test

Approximately half of the HIV positive MDR-TB patients in each study were already on ART prior to MDR-TB treatment initiation (see Additional file 5). The overall proportion of MDR-TB and HIV positive patients on ART was high except in two studies [16, 23], pooled proportion 83% (see Additional file 6). Treatment success varied between HIV negative and positive MDR-TB patients, with higher rates in HIV negative patients, in all but one study [13]. Unsuccessful treatment in contrast was higher among HIV positive cases compared to HIV negative cases in five studies [15–17, 22, 24], but was lower in one study [13]. The ratio of treatment success to unsuccessful treatment was about 2:1 among HIV positive patients and 3:1 among HIV negative patients (see Additional file 5). The pooled risk ratio for successful MDR-TB treatment in HIV positive versus HIV negative patients was 0.87 (95% CI, 0.79, 0.96) (Fig. 2).

### Cure according to ART status

Uptake of ART for HIV did not affect the proportion whose MDR-TB was cured among the MDR-TB and HIV co-infected patients. Cure outcomes ranged from; 28.6 to 54.7% among patients on ART, and from 22.2 to 57.7% among those not on ART medication (Table 1).

**Table 1:** Cure rates for MDR-TB and HIV coinfected patients in SSA by ART status

Author and year	On ART		No ART	
	Cured (%)	Not cured (%)	Cured (%)	Not cured (%)
Umanah et al 2015 [14]	312 (32.9)	635 (67.1)	NR	NR
Van der Walt et al 2016 [16]	52 (54.7)	43 (45.3)	172 (57.7)	126 (42.3)
Mugabo et al 2015 [23]	19 (35)	35 (65)	14 (34)	27 (66)
Padayatchi et al 2014 [25]	4 (28.6)	10 (71.4)	2 (22.2)	7 (77.8)
Umanah et al 2015_b_ [24]	339 (29.8)	798 (70.2)	NR	NR

*NR* Not reported.

## Discussion

This review identified nine studies, reporting data on treatment of MDR-TB among those living with HIV in SSA. The most frequently reported outcome for MDR-TB was cure, followed by death and the least commonly reported outcome was still being on treatment for MDR-TB. Overall, HIV ART uptake was high among those MDR-TB patients living with HIV, however MDR-TB treatment was still less successful among this group than among those not HIV co-infected.

The most common MDR-TB treatment outcome among the MDR-TB and HIV co-infected patients was cure (pooled proportion 34.9%). This is higher than the reported proportion cured (16%) among MDR-TB and HIV co-infected patients in WHO region Europe [26]. The low cure proportion in the WHO region Europe, could be attributed to the variation in quality of MDR-TB and HIV treatment services within different parts of Europe, with patients in Eastern Europe more likely to receive empiric anti-TB treatment with reduced activity compared to other parts of Europe. There were also only a small number of MDR-TB and HIV patients involved in the study which might have skewed the outcome [27, 28]. In addition, the HIV epidemic in Europe is a concentrated one, focused on higher risk groups including people who inject drugs, whilst that in SSA is as generalised epidemic.

High levels of death and default accounted for unsuccessful outcomes in this study. Unsuccessful treatment was higher in MDR-TB patients living with HIV than in other MDR-TB patients. Poorer treatment outcomes have also been reported in a review of outcomes among MDR-TB patients living with HIV and other comorbidities [19]. These findings indicate the need for increased efforts to improve management of MDR-TB and HIV co-infection, such as, active case holding for immediate initiation on therapy and active mechanisms for tracing those who default. However, higher levels of unsuccessful treatment among MDR-TB patients with HIV compared to those without HIV observed in this study, contradicts the findings from previous reviews that report similar levels of MDR-TB treatment success in both groups [6, 7]. These reviews included studies from settings with a low prevalence of HIV which might have impacted their overall findings related to effect of HIV status.

Observed mortality was high in this review at 18.1% (studies ranging from 11.2 to 34.3%), whilst lower than 38% pooled mortality in a similar global review on adults and children [6], it was higher than the 12% mortality reported in the WHO region Europe [26] and 11% in a review on impact of ART on mortality among TB and HIV patients [29]. A lower mortality than the global review could be due to high ART uptake in the studies included in our review, however higher mortality than that reported in the other two reviews may be related to differences in the populations affected and regional differences in the epidemiology of HIV and MDR-TB.

In this review, the pooled proportion of patients who defaulted was 6.8%. This was lower than 16.1% [6] reported in a previous review. Worst outcomes in the previous review were reported among early cohorts prior to ART roll out when the use of ART was limited. In addition, some studies in our review did not report default outcomes [15, 17, 25] which might have affected the pooled value.

Though centralised treatment settings have been praised for their high treatment retention and low levels of default [30], the higher levels of defaults in this review were identified in studies in centralised settings. However, a review by Weiss et al [31] demonstrated no difference in outcome between treatment settings. More investigations are required to understand program related characteristics in both settings that might have produced this observation.

The uptake of ART among those living with HIV increased during the course of MDR-TB treatment, from approximately 50% uptake prior to treatment to an overall pooled uptake of 83% (0.83). Progressive increase in ART uptake during MDR-TB and HIV co-infection treatment, illustrates active implementation of the WHO recommendation for concurrent treatment in MDR-TB and HIV co-infection [11, 22]. ART uptake in this review is high compared to an uptake of 67% among MDR-TB and HIV co-infected patients in Eastern Europe. This is attributed to the weak implementation of concurrent treatment in Eastern Europe, following a rise in HIV prevalence [32].

Ideally, we would expect high ART uptake to improve treatment outcomes for MDR-TB and HIV co-infected patients. This was not the case in this study. Poor adherence to ART and the complication in the management of MDR-TB and HIV co-infection maybe responsible [14, 33]. In addition to opportunistic infections, most MDR-TB and HIV co-infected patients included in retained studies, reported low CD4 cells < 100 cells/mm^3^, being severely underweight and having chest cavity lesions at baseline [15, 17]. Patients with CD4 counts < 100 cells/mm^3^ are more likely to die after starting treatment [14, 23]. Therefore, the poor outcomes observed in this proportion of patients may not be attributed solely to the ineffectiveness of the treatment program but partially to the baseline characteristic of these patients prior to treatment.

### Limitation

Our review has a number of limitations. We assumed that patients who default therapy or who are lost to follow-up during TB treatment, will have done so because they were experiencing poor outcomes. Therefore, default, lost to follow up, on treatment and transfer out, were classified into unsuccessful treatment in the analysis. One study had children aged under 15 years nested within the study population [23]. However, there were only 7 MDR-TB and HIV co-infected children within the 363 participants. We assumed their effect on the analysis was negligible. One study had a sample size of 23 participants [25], this randomised control trial had methodological shortcomings and was rated as poor quality. This study was dropped from subgroup analysis of MDR-TB patients according to HIV status. However data from this study was included in the tables, and in the forest plots on treatment outcomes and proportion of ART uptake, so as to present the contribution of each study. Inclusion of this study generated high heterogeneity scores. There were limited data to examine the impact of ART uptake among MDR-TB patients living with HIV, this probably reflecting WHO recommendations [11] for concurrent treatment for all co-infected patients. This limited our ability to investigate the impact of ART on MDR-TB treatment outcomes. Not all studies provided data on all items of interest which could lead to bias. Eight of nine studies were from Southern African (Bostwana, Lesotho and South Africa) and one was from East Africa (Ethiopia), possibly reflecting this review only including studies published in English. The findings should therefore, only be generalised to the entire SSA region with caution.

The paucity of data from outside of southern Africa is a concern and may reflect limited research resources available among west, central and east African countries. Lack of infrastructure, equipment, human resources and absence of good health information system [5, 18] in TB programs have been advanced as reasons for paucity in MDR-TB data and studies conducted in SSA. The number of publications on treatment outcomes for MDR-TB and HIV coinfection in SSA are low and the quality of studies is generally weak when compared to those in similar global reviews [6, 18, 32]. This reiterates the need for more research in SSA region.

## Conclusion

Despite endemicity of HIV in SSA, treatment outcomes for MDR-TB and HIV co-infected patients on ART do not vary widely from those reported globally. However, treatment success rates are lower in HIV positive patients compared to HIV negative patients. This is probably due to high levels of deaths and defaults in HIV positive patients. ART uptake was high with progressive increase in uptake as MDR-TB and HIV treatment advances. Unsuccessful treatment outcome and low cure rates in patients on ART most probably reflect poor adherence to therapy. Emphasis should be laid on prompt initiation, support of patients, and ART adherence, to reduce the debilitating effect of low CD4 counts and opportunistic infections that may increase chances of death and other unfavourable outcomes. These findings indicate that the DOTS plus strategy should be strengthened alongside scaling up of integrated decentralized treatment strategies.

## Additional files

Additional file 1: Treatment outcomes for MDRTB-HIV patients. Following the proposal by Learson et al 2005 and WHO, 2008 (DOCX 13 kb)

Additional file 2: Summary of study characteristics included in the review. This provides a breakdown of all studies included in the review. (DOCX 17 kb)

Additional file 3: Treatment outcome for all MDR-TB patients. This file displays the proportion of treatment outcomes for MDRTB-HIV co-infected patients among included studies. (DOCX 16 kb)

Additional file 4: Proportion of MDRTB-HIV patients who were cured and those who died of MDR-TB in SSA. Forest plots illustrating proportion of MDRTB-HIV patients cured and those who died during MDR-TB treatment. (DOCX 6176 kb)

Additional file 5: HIV ART uptake and treatment success for MDR-TB by HIV status in SSA. This file provides a breakdown of ART uptake prior to and during treatment for MDRTB-HIV co-infected patients in SSA. It also compares treatment success between HIV positive and HIV negative MDR-TB patients. (DOCX 15 kb)

Additional file 6: Proportion of ART uptake in the context of MDRTB-HIV co-infection in SSA. Forest plot illustrating the proportion of ART uptake among MDRTB-HIV co-infected patients in SSA. (DOCX 6176 kb)

Additional file 7: Search strategy. This file demonstrates how database search was conducted. (DOCX 13 kb)

Additional file 8: Sample data extraction form. This file illustrates how the data extraction form was designed (DOCX 25 kb)

## Data Availability

All relevant data for this study have been added as additional files .

## Abbreviations

ART: Antiretroviral therapy
CD4: Cluster of Differentiation 4
DOTS: `Direct Observed Treatment, Short course
MDR-TB: Multidrug resistant TB
MDRTB-HIV: Multidrug resistant tuberculosis-HIV coinfection
RR: Risk Ratio
SLD: Second Line anti tuberculosis Drug
SSA: Sub Saharan Africa
TB: Tuberculosis
