# Supplementary material for: Treatment outcomes and antiretroviral uptake in multidrug-resistant tuberculosis and HIV co-infected patients in Sub Saharan Africa: a systematic review and meta-analysis
Source: BMC Infect Dis. 2019 Aug 16;19:723. doi: 10.1186/s12879-019-4317-4 (PMC6697933; doi:10.1186/s12879-019-4317-4)
Supplement: Supplementary file 1 — Treatment outcomes for MDRTB-HIV patients. Following the proposal by Learson et al 2005 and WHO, 2008 (DOCX 13 kb) [file 12879_2019_4317_MOESM1_ESM.docx]

**Additional file 1: Treatment outcomes for MDRTB-HIV patients**

| *Cure* | Treatment completed as recommended by the national policy without evidence of failure and three or more consecutive cultures taken at least 30 days apart, all with negative results after the intensive phase |
| --- | --- |
| *Treatment completed* | Treatment completed as recommended by the national policy without evidence of failure but no record that three or more consecutive cultures taken at least 30 days apart are negative after the intensive phase |
| *Treatment failure* | Treatment terminated or need for permanent regimen change of at least two anti-TB drugs because of: lack of conversion by the end of the intensive phase, or bacteriological reversion in the continuation phase after conversion to negative, or evidence of additional acquired resistance to fluoroquinolones or second-line injectable drugs, or adverse drug reactions |
| *Died* | A patient who dies for any reason during treatment |
| *Lost to follow-up* | A patient whose treatment was interrupted for 2 consecutive months or more |
| *Not evaluated* | A patient for whom no treatment outcome is assigned. This includes cases “transferred out” to another treatment unit and whose treatment outcome is unknown |
| *Treatment success* | The sum of cured and treatment completed |
