# Supplementary material for: Treatment outcomes and antiretroviral uptake in multidrug-resistant tuberculosis and HIV co-infected patients in Sub Saharan Africa: a systematic review and meta-analysis
Source: BMC Infect Dis. 2019 Aug 16;19:723. doi: 10.1186/s12879-019-4317-4 (PMC6697933; doi:10.1186/s12879-019-4317-4)
Supplement: Supplementary file 2 — Summary of study characteristics included in the review. This provides a breakdown of all studies included in the review. (DOCX 17 kb) [file 12879_2019_4317_MOESM2_ESM.docx]

| **Author** | **Study period** | **Country** | **Study design** | **Study description** | **Sample size** | **MDRTB-HIV co-infected (%)** | **Number of HIV positives on ART (%)** | **Type of TB** | **Treatment regimen** | **Treatment setting** | **TB drugs used for treatment** | **ART used** | **Treatment duration (months)** |
| --- | --- | --- | --- | --- | --- | --- | --- | --- | --- | --- | --- | --- | --- |
| Umanah et al 2015 ^15^ | 2007-2010 | South Africa | Retrospective cohort | A review of the effect of time of ART initiation on treatment outcomes | 947 | 947 (100) | 947 (100) | PTB&EPTB | Mixed | Centralised | KAN, MOXIF, ETHIO, TERI, ETHAM, PYRA | **NR** | **24** |
| Satti et al 2012 ^14^ | 2008-2009 | Lesotho | Retrospective cohort | Review of medical charts to report outcomes of MDRTB and HIV treatment | 134 | 94 (70.14) | 90 (98.7) | PTB&EPTB | standardised | Decentralised | KAN, PYRA, LEVO, PROTH, CYCLO, PASA | EFA, LAM, ZID | **22.9** |
| Meressa et al 2015 ^16^ | 2009-2014 | Ethiopia | Retrospective cohort | Clinical outcomes for MDRTB with 24months follow up SLD treatment | 612 | 133 (21.73) | 131(98.5) | PTB&EPTB | Standardised | Decentralised | LEVO, ETHIO, CYCLO, PASA, PYRA, KAN, AMIK, CAPREO | EFA, LAM, TEN, STAV ZID | **24** |
| Van der Walt et al 2016 ^17^ | 2000-2008 | South Africa | Retrospective cohort | Investigating causes of death among MDRTB, contributions of other factors and HIV | 671 | 393 (58.56) | 95(24.2) | NR | Standardised | Centralised | **NR** | **NR** | **22+** |
| Brust et al 2018 ^21^ | 2011-2013 | South Africa | prospective cohort | comparison of survival and treatment outcomes in MDRTB-HIV patients on ART to MDR TB patients alone | 206 | 150 (72.25) | 138(96.0) | PTB&EPTB | Standardised | Centralised | KAN, MOXI, ETHIO, TERI, ETHAM, PYRA, | EFA, LAM, STAV, TEN | **24** |
| Shin et al 2017 ^18^ | 2006-2013 | Botswana | prospective cohort | Treatment outcomes among HIV+ and HIV- MDR-TB patients after the expansion of the ART program | 588 | 408 (69.38) | 351(86.0) | NR | Standardised | Decentralised | AMIK, LEVO, ETHIO, CYCLO, PYRA | **NR** | **18+** |
| Mugabo et al 2015 ^25^ | 2004-2006 | South Africa | Retrospective Case-control | Investigating whether HIV and ART drugs influence MDR-TB treatment outcomes | 363 | 95 (26.17) | 54(56.8) | NR | Individualised | Centralised | KAN, ETHIO, OFL, TERI, PYRA, ETHAM | EFA, LAM, STAV | **18+** |
| Umanah et al 2015_b_ ^26^ | 2007-2010 | South Africa | Retrospective cohort | Investigating predictors of cure among MDR-TB HIV co-infected patients | 1137 | 1137 (100) | 1137(100) | PTB&EPTB | Mixed | Centralised | KAN, MOXIF, ETHIO, TERI, ETHAM, PYRA | **NR** | **24** |
| Padayatchi et al 2014 ^24^ | 2008-2012 | South Africa | RCT | Assessed therapeutic outcomes of ART integration with MDR-TB treatment | 23 | 23 (100) | 17(73.9) | PTB&EPTB | Standardised | Centralised | **NR** | DIDA, EFA,  LAMI | **18** |

**Additional file 2: Summary of study characteristics included in the review**

NR: Note reported. RCT: Randomised Controlled Trial. PTB: Pulmonary TB. EPTB: Extra-pulmonary TB. KAN: Kanamycin. MOXIF: Moxifloxacine. ETHIO: Ethionamide. TERI: Terizidone. ETHAM: Ethambutol. PYRA: Pyrazinamide. LEVO: Levofloxacin. PROTH: Prothionamide. CYCLO: Cycloserine. PASA: Para-aminosalysilic acid. AMIK: Amikacin. CAPREO: Capreomycin. OFL: Ofloxacin. EFA: Efavirenz. LAM: Lamivudine. ZID: Zidovudine. TEN: Tenofovir. STAV: Stavudine. DIDA: Didanosine.
