# Supplementary material for: Treatment outcomes and antiretroviral uptake in multidrug-resistant tuberculosis and HIV co-infected patients in Sub Saharan Africa: a systematic review and meta-analysis
Source: BMC Infect Dis. 2019 Aug 16;19:723. doi: 10.1186/s12879-019-4317-4 (PMC6697933; doi:10.1186/s12879-019-4317-4)
Supplement: Supplementary file 3 — Treatment outcome for all MDR-TB patients. This file displays the proportion of treatment outcomes for MDRTB-HIV co-infected patients among included studies. (DOCX 16 kb) [file 12879_2019_4317_MOESM3_ESM.docx]

**Additional file 3:** Treatment outcome for all MDRTB patients (proportion [%] for MDRTB-HIV co-infected cases)

| Author and Year | Cure | Completed treatment | Died | Lost to follow up | Default | On treatment | Transfer out | Failed |
| --- | --- | --- | --- | --- | --- | --- | --- | --- |
| Umanah *et al* 2015 ^15^ | 312 (32.9) | 163 (17.2) | 181 (19.1) | **NR** | 206 (21.8) | 13 (1.4) | 44 (4.7) | 28 (3) |
| Satti *et al* 2012 ^14^ | 71 (52.9) | 12 (9.0) | 46 (34.3) | **NR** | 1 (1) | **NR** | 3 (2.2) | 1 (1) |
| Meressa *et al* 2015 ^16^ | 396 (64.7) | 85 (13.9) | 85 (13.9) | 36 (5.9) | **NR** | **NR** | **NR** | 10 (1.6) |
| Van der Walt *et al* 2016 ^17^ | 436 (65) | | 158 (23.5) | 2 (0.3) | 33 (4.9) | **NR** | 28 (4.1) | 14 (2.1) |
| Brust *et al* 2018 ^21^ | 130 (68) | 10 (5.2) | 22(11.2) | **NR** | 23 (12.0) | **NR** | **NR** | 6 (3.1) |
| Shin *et al* 2017 ^18^ | 177 (30.1) | 264 (44.9) | 118 (20.1) | 24 (4.1) | **NR** | **NR** | **NR** | 5 (0.9) |
| Mugabo *et al* 2015 ^25^ | 131 (36.1) | 61(16.8%) | 45 (12.4) | **NR** | 76 (21) | **NR** | 21 (5.8) | 29 (8.0) |
| Umanah *et al* 2015_b_ ^26^ | 339 (29.8) | 188 (16.5) | 258 (22.7) | **NR** | 254 (22.3) | 13 (1.1) | 52 (4.6) | 33(2.9) |
| Padayatchi *et al* 2014 ^24^ | 6 (26.1) | **NR** | 7 (30.4) | 4 (17.4) | **NR** | 6 (26.1) | **NR** |  |
| *% Summary* | ***34.9*** | ***9.9*** | ***18.1*** | ***4.0*** | ***6.8*** | ***2.3*** | ***3.6*** | ***2.1*** |
| *Confidence Interval* | ***25.3, 46.3*** | ***5.3,17.9*** | ***13.8,23.3*** | ***0.4,3.4*** | ***3.7,12.4*** | ***0.5, 3.4*** | ***1.2, 3.8*** | ***1.4,3.3*** |
| *I^2^* | ***98.9*** | ***96.6*** | ***89.6*** | ***82.7*** | ***93.5*** | ***85.3*** | ***68.1*** | ***53.0*** |
| *P value* | ***0.010*** | ***0.000*** | ***0.00*** | ***0.00*** | ***0.00*** | ***0.00*** | ***0.00*** | ***0.00*** |

NR: Note reported. % Summary: Pooled proportion for MDRTB-HIV coinfected patients
