# Supplementary material for: Treatment outcomes and antiretroviral uptake in multidrug-resistant tuberculosis and HIV co-infected patients in Sub Saharan Africa: a systematic review and meta-analysis
Source: BMC Infect Dis. 2019 Aug 16;19:723. doi: 10.1186/s12879-019-4317-4 (PMC6697933; doi:10.1186/s12879-019-4317-4)

**Additional file 4:** Proportion of MDRTB-HIV patients who were cured and those who died of MDRTB in SSA. CI= confidence interval, ES = Effect estimate


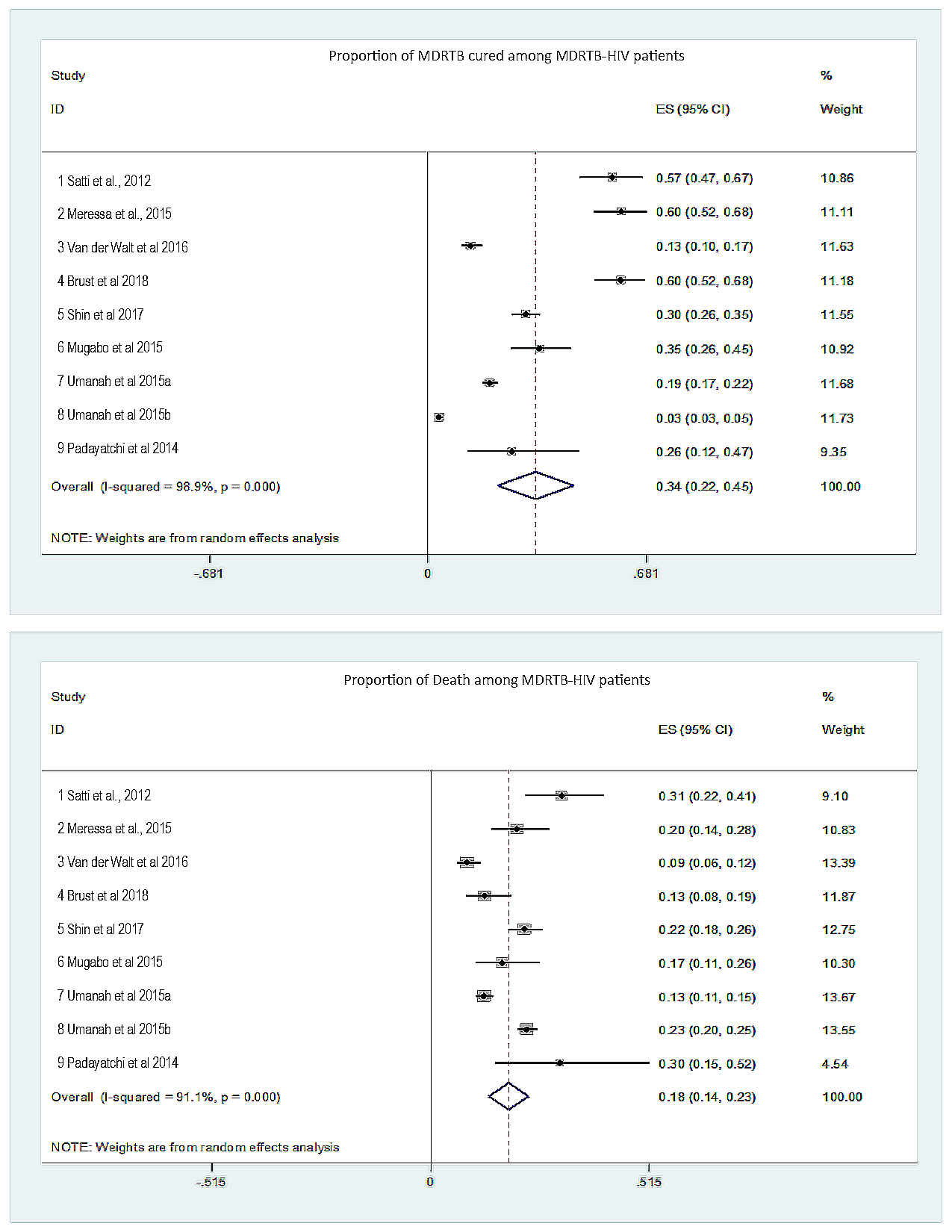

Supplement: Supplementary file 4 — Proportion of MDRTB-HIV patients who were cured and those who died of MDR-TB in SSA. Forest plots illustrating proportion of MDRTB-HIV patients cured and those who died during MDR-TB treatment. (DOCX 6176 kb) [file 12879_2019_4317_MOESM4_ESM.docx]
