# Supplementary material for: Treatment outcomes and antiretroviral uptake in multidrug-resistant tuberculosis and HIV co-infected patients in Sub Saharan Africa: a systematic review and meta-analysis
Source: BMC Infect Dis. 2019 Aug 16;19:723. doi: 10.1186/s12879-019-4317-4 (PMC6697933; doi:10.1186/s12879-019-4317-4)
Supplement: Supplementary file 5 — HIV ART uptake and treatment success for MDR-TB by HIV status in SSA. This file provides a breakdown of ART uptake prior to and during treatment for MDRTB-HIV co-infected patients in SSA. It also compares treatment success between HIV positive and HIV negative MDR-TB patients. (DOCX 15 kb) [file 12879_2019_4317_MOESM5_ESM.docx]

**Additional file 5: HIV ART uptake and treatment success for MDRTB by HIV status in SSA**

| Author  And Year |  |  | HIV positive | | HIV negative | |
| --- | --- | --- | --- | --- | --- | --- |
|  | **% ART uptake** | **% on ART prior to MDRTB treatment** | **Treatment success rates ( per 1000)** | **Treatment unsuccessful rates ( per 1000)** | **Treatment success rates ( per 1000)** | **Treatment unsuccessful rates ( per 1000)** |
| Umanah *et al* 2015 ^15^ | 100.00 | 57.55 | 501.58 | 498.42 | **NR** | **NR** |
| Satti *et al* 2012 ^14^ | 95.74 | 55.56 | 659.57 | 340.43 | 525.00 | 475.00 |
| Meressa *et al* 2015 ^16^ | 98.50 | 91.60 | 699.25 | 300.75 | 810.02 | 189.98 |
| Van der Walt *et al* 2016 ^17^ | 24.17 | 96.84 | 570.00 | 430.00 | 762.59 | 237.41 |
| Brust *et al* 2018 ^21^ | 92.00 | 84.03 | 702.90 | 297.10 | 811.32 | 188.68 |
| Shin *et al* 2017 ^18^ | 86.3 | 59.26 | 730.39 | 269.61 | 794.44 | 205.56 |
| Mugabo *et al* 2015 ^25^ | 100.00 | 57.55 | 463.50 | 536.50 | **NR** | **NR** |
| Umanah *et al* 2015_b_ ^26^ | 56.84 | NR | 473.68 | 526.32 | 548.51 | 451.49 |
| Padayatchi *et al* 2014 ^24^ | 70.63 | 0.00 | 260.87 | 739.13 | **NR** | **NR** |

NR: Note reported.
