# Supplementary material for: Treatment outcomes and antiretroviral uptake in multidrug-resistant tuberculosis and HIV co-infected patients in Sub Saharan Africa: a systematic review and meta-analysis
Source: BMC Infect Dis. 2019 Aug 16;19:723. doi: 10.1186/s12879-019-4317-4 (PMC6697933; doi:10.1186/s12879-019-4317-4)

**Additional file 6:** Proportion of ART uptake in the context of MDRTB-HIV coinfection in SSA. CI = Confidence Interval ES = Effect


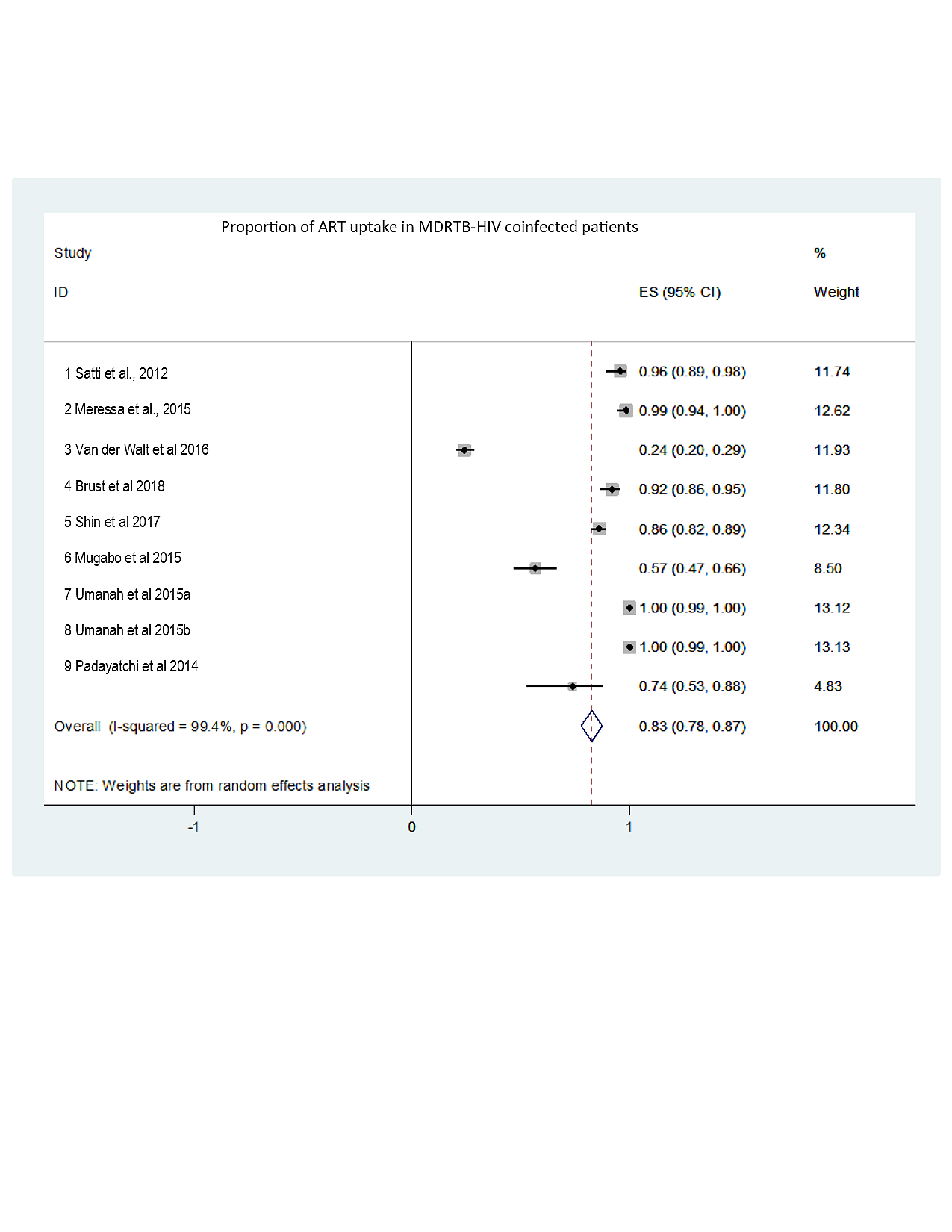

Supplement: Supplementary file 6 — Proportion of ART uptake in the context of MDRTB-HIV co-infection in SSA. Forest plot illustrating the proportion of ART uptake among MDRTB-HIV co-infected patients in SSA. (DOCX 6176 kb) [file 12879_2019_4317_MOESM6_ESM.docx]
