# Supplementary material for: Treatment outcomes and antiretroviral uptake in multidrug-resistant tuberculosis and HIV co-infected patients in Sub Saharan Africa: a systematic review and meta-analysis
Source: BMC Infect Dis. 2019 Aug 16;19:723. doi: 10.1186/s12879-019-4317-4 (PMC6697933; doi:10.1186/s12879-019-4317-4)
Supplement: Supplementary file 7 — Search strategy. This file demonstrates how database search was conducted. (DOCX 13 kb) [file 12879_2019_4317_MOESM7_ESM.docx]

## **Additional file 7: Search strategy**

Search Strategy (Medline EbscoHost), conducted between February to May 2018

|  | **Search terms** | **Results** |
| --- | --- | --- |
| S1 | Treatment OR Treat OR Treatment outcom* OR Outcom* OR Survival OR Success OR Failur* OR (MH “Treatment outcome”) OR (MH “Withholding treatment”) OR (MH “Treatment refusal”) OR (MH “Treatment failure”) OR (MH “Residential outcome”) OR Therapy | 8,285,709 |
| S2 | Tuberculosis, Multidrug-Resistan* OR Multidrug resistant TB OR Multidrug resistant Tuberculosis OR MDR Tuberculosis OR MDRTB OR MDR TB OR MDR-TB OR (MH “Tuberculosis Multidrug resistant”) OR Tuberculosis | 239 272 |
| S3 | HIV OR AIDS OR Human Immunodeficiency Virus OR Human immune deficiency virus OR Human immune-deficiency virus OR People living with HIV OR PLWH OR (MH “HIV”) OR (MH “HIV Long term survivors”) OR (MH “HIV Long Terminal Repeat”) OR (MH “HIV Long term seropositivity”) | 423,646 |
| S4 | Antiretroviral OR Antiretroviral therapy OR Anti-retroviral OR Anti-retroviral therapy OR ART OR ARV OR Antiretroviral medication OR Antiretroviral regimen OR Antiretroviral administration OR Highly active antiretroviral OR (MH “Art”) OR (MH “Art Therapy”) | 148,987 |
| S5 | Sub Saharan Africa OR Africa South of Sahara OR Saharan Africa OR Africa OR Angola OR Benin OR Botswana OR Burkina Faso OR Burundi OR Cameroon OR Cape Verde OR Central African Republic OR Chad OR Comoros OR Congo (Brazzaville) OR Congo (Democratic Republic) OR Côte d'Ivoire OR Djibouti OR Equatorial Guinea OR Eritrea OR Ethiopia OR Gabon OR The Gambia OR Ghana OR Guinea OR Guinea-Bissau OR Kenya OR Lesotho OR Liberia OR Madagascar OR Malawi OR Mali OR Mauritania OR Mauritius OR Mozambique OR Namibia OR Niger OR Nigeria OR Réunion OR Rwanda OR Sao Tome and Principe OR Senegal OR Seychelles OR Sierra Leone OR Somalia OR South Africa OR Sudan OR Swaziland OR Tanzania OR Togo OR Uganda OR Western Sahara OR Zambia OR Zimbabwe | 514,826 |
| S6 | S1 AND S2 AND S3 AND S4 AND S5 | 115 |
| S7 | S1 AND S2 AND S4 AND S5 | 116 |
| S8 | S1 AND S2 AND S3 AND S5 | 565 |
