# Supplementary material for: Treatment outcomes and antiretroviral uptake in multidrug-resistant tuberculosis and HIV co-infected patients in Sub Saharan Africa: a systematic review and meta-analysis
Source: BMC Infect Dis. 2019 Aug 16;19:723. doi: 10.1186/s12879-019-4317-4 (PMC6697933; doi:10.1186/s12879-019-4317-4)
Supplement: Supplementary file 8 — Sample data extraction form. This file illustrates how the data extraction form was designed (DOCX 25 kb) [file 12879_2019_4317_MOESM8_ESM.docx]

## **Additional file 8: Sample data extraction form**
